# Investigation of Potting-Adhesive-Induced Thermal Stress in MEMS Pressure Sensor

**DOI:** 10.3390/s21062011

**Published:** 2021-03-12

**Authors:** Yunfan Zhang, Bowen Li, Hui Li, Shengnan Shen, Feng Li, Wentao Ni, Wan Cao

**Affiliations:** 1 The Institute of Technological Sciences, Wuhan University, Wuhan 430072, China; zhang_yunfan@whu.edu.cn (Y.Z.); shen_shengnan@whu.edu.cn (S.S.); 2 School of Power and Mechanical Engineering, Wuhan University, Wuhan 430072, China; li_bowen@whu.edu.cn (B.L.); ni_wentao@whu.edu.cn (W.N.); 3 School of Electrical and Electronic Engineering, Wuhan Polytechnic University, Wuhan 430023, China; lifeng20210719@163.com; 4 Wuhan FineMEMS Inc., Wuhan 430075, China; wcao@finemems.com

**Keywords:** MEMS pressure sensor, potting adhesive, thermal stress, output error

## Abstract

Thermal stress is one of the main sources of micro-electro-mechanical systems (MEMS) devices error. The Wheatstone bridge is the sensing structure of a typical piezoresistive MEMS pressure sensor. In this study, the thermal stress induced by potting adhesive in MEMS pressure sensor was investigated by experiments, calculated by analytics and analyzed by simulations. An experiment system was used to test the sensor at different air pressures and temperatures. The error becomes greater with the decrease in pressure. A set of novel formulas were proposed to calculate the stress–strain on Wheatstone bridge. The error increases with the temperature deviating from 25 °C. A full-scale geometric model was developed, and finite element simulations were performed, to analyze the effect of the stress on MEMS pressure sensor induced by different temperatures and thicknesses of potting adhesive. Simulation results agree well with the experiments, which indicated that there is a 3.48% to 6.50% output error in 0.35 mm potting adhesive at 150 °C. With the thickness of potting adhesive increasing, the variations of output error of the Wheatstone bridge present an N-shaped curve. The output error meets a maximum of 5.30% in the potting adhesive of 0.95 mm and can be reduced to 2.47%, by increasing the potting adhesive to 2.40 mm.

## 1. Introduction

The micro-electro-mechanical systems (MEMS) pressure sensor is the first industrial MEMS device in the world which was micromachined by Honeywell in 1962 [1]. With the development of electronic processing technology, MEMS pressure sensors have been widely used in industrial productions [2], consumer electronics [3] and vehicles [4]. There are many pressure sensor manufacturers, such as Bosch (Germany), Honeywell (USA) and DENSO (Japan) They keep optimizing the processes to improve the accuracy and reduce the error of pressure sensor.

Process residual stress is one of the main factors in the performance of the pressure sensor. Scientists have done a lot of work to reduce residual stress. Some tried to design new structures, to reduce the output error induced by residual stress. Zhang et al. [5] introduced a novel plastic packaging for MEMS pressure sensor which suggested that the adhesive should be chosen for lower thickness and larger Young’s modulus to make better stability. Li et al. [6] designed a novel piezoresistive pressure sensor with a four-beams-bossed-membrane structure which could improve both sensitivity and linearity. Wang et al. [7] introduced an acoustic pressure sensor with an integrated vacuum cavity that could measure pressure without an external package. Tran et al. [8] designed a novel MEMS piezoresistive pressure sensor for low-pressure measurements which had four independent petal membranes. This structure increased the sensitivity and decreased the nonlinearity of the sensor. These novel designs improved the performance of pressure sensor, but the scientists did not study the mechanism of output error. However, there are other scientists who have done works on it. Krondorfer et al. [9] investigated the stress from the package which caused asymmetric bending deformations on the silicon membrane and resulted in an imbalance of the Wheatstone bridge. Marina et al. [10] analyzed the process-induced residual stresses by a virtual prototyping approach, to study the effect of the process parameters, which showed the distribution of residual stresses caused by the adhesive during the curing. Wu et al. [11] investigated the thermal stress on two sets of electronic control units by moiré interferometry. It showed worse accuracy when the sensors were heated. Zhang et al. [12] studied the output error of inertial measurement unit caused by acoustic injection. Tran et al. [13] investigated the effect of temperature on the output of a piezoresistive sensor and concluded that the thermal performance instability is mainly attributable to the effects of temperature, the residual stress due to fabrication and the residual stress due to packaging. Chiou et al. [14] proposed a methodology that can be used to predict the thermal hysteresis and further improve the sensing element design. Lu et al. [15] studied the thermal stress analysis of chip by finite element model with variations of material properties and geometric parameter of adhesive. Xu et al. [16] used the finite element method to predict the performance of a piezoresistive transducer pressure sensor to thermal and pressure environments. Subbiah [17] characterized the influence of thermal stresses in the sensor and produced a final version of the sensor that is stable for operations at high temperature. Zhang et al. [18] studied the influence of material parameters of bonding adhesive on the performance of piezoresistive pressure sensor. With a lower Young’s modulus bonding adhesive, there are lower residual stress and smaller zero offset to the sensor. Chou et al. [19] investigated thermal and packing effects on the sensitivity of pressure sensor. There are large differences of thermal stress at different geometry of protection adhesive, and the uneven type is suggested to reduce thermal effect. Andreas et al. [20] studied the time-dependent hysteresis effects of pressure sensor which cause stresses and lead to signal error. A numerical model was developed to reproduce the sensor behaviors and design the properties of adhesive. The previous studies only focus on novel structures and different material parameters of adhesive but fail to reveal the influence of potting adhesive on the thermal-stress-induced output error of Wheatstone bridge in MEMS pressure sensors.

This paper quantitatively studies the thermal stress–strain induced by potting adhesive in MEMS pressure sensor, which was investigated by experiments, calculated by analytics and analyzed by simulations. An experiment system was used to study the output error of the MEMS pressure sensor at working pressures and temperatures. A set of novel formulas were proposed to study the mechanical effects of thermal stress by calculating the stress–strain on Wheatstone bridge. A full-scale geometric model was developed, and finite element simulations were performed, to optimize the potting process parameter by analyzing the output error of the sensor induced by different temperatures and thicknesses of potting adhesive.

## 2. Experimental Investigation

**Figure 1:**
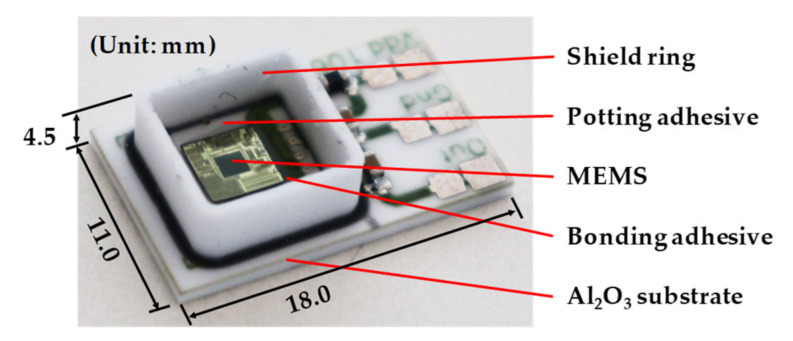
MEMS pressure sensor.

**Figure 2:**
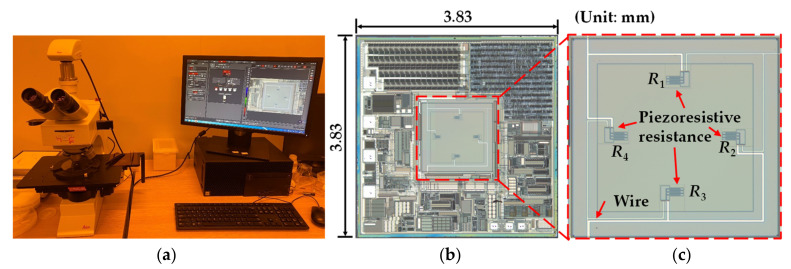
(**a**) Optical microscope for taking pictures of MEMS pressure chip. (**b**) MEMS pressure chip. (**c**) Wheatstone bridge.

A typical piezoresistive MEMS pressure sensor was tested to analyze the output voltage error at different air pressures and temperatures. The research object in this paper is shown in Figure 1. It is a pressure sensor (FN-FPT1065, FineMEMS, Wuhan, China) which contains a piezoresistive MEMS pressure chip (MLX90808, Melexis, Tessenderlo, Belgium). It can be used in the environment of −40 to 150 °C and can detect a maximum pressure of 100 kPa. The Wheatstone bridge resistance of MLX90807 is 10 kΩ. The sensor was calibrated at 25 °C and 0 kPa external pressure. The actual output contains both detecting data and error. Figure 1 shows the sensor without an outside package shell. Its dimension is 18.0 × 11.0 × 4.5 mm, which is mainly composed of five parts: shield ring, potting adhesive, MEMS, bonding adhesive and Al_2_O_3_ substrate. The MEMS is bonded on the Al_2_O_3_ substrate by a bonding adhesive. The shied ring is set to surround the MEMS, to keep the potting adhesive which covers the MEMS chip. For better observation, an optical microscope (DM2500, Leica, Germany) was used to take pictures of the Wheatstone bridge, as shown in Figure 2a. The whole structure of the MEMS pressure chip is shown in Figure 2b whose dimension is 3.83 × 3.83 × 0.55 mm. The Wheatstone bridge is shown in Figure 2c, and it is composed of 4 piezoresistive resistances and wires.

**Figure 3:**
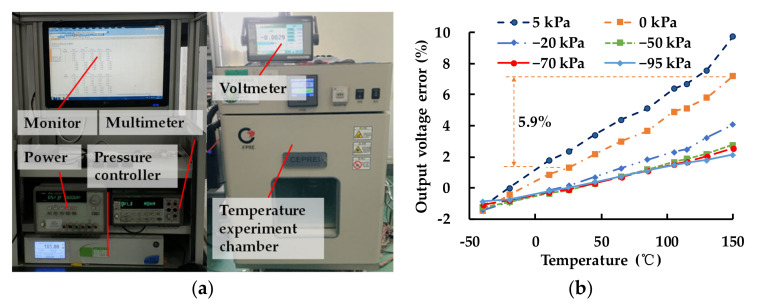
(**a**) MEMS pressure-sensor testing system; (**b**) output voltage errors of the MEMS pressure sensor at different air pressures and temperatures.

The testing system is made of a pressure controller, temperature experiment chamber and electrical instruments (voltmeter, multimeter, etc.), which are shown in Figure 3a. The sensor was tested at different temperatures: −40, −20, 10, 25, 45, 65, 85, 105, 115, 130 and 150 °C. In the meantime, the air pressures loaded on the sensor were changed into 5, 0, −20, −50, −70 and −95 kPa (the downward pressure is positive, and the upward pressure is negative). A 5 V voltage was supplied to the testing sensor.

The output voltage errors of the MEMS pressure sensor are shown in Figure 3b. The sensor was calibrated at 25 °C. It can be found that the output voltage errors become larger with the temperature increasing. There was a 2.15% maximum error when the sensor was tested from −40 to 150 °C, at the pressure of −95 kPa, a 2.63% error at −70 kPa and a 2.92% error at −50 kPa. When the pressures were lower, the output voltage error got worse. There was a 3.94% error at −20 kPa, 5.90% error at 0 kPa and 7.40% error at 5 kPa. The testing results show that thermal stress has a significant effect on the MEMS pressure sensor and the sensor is more affected under low pressure.

## 3. Analytic Analysis

**Figure 4:**
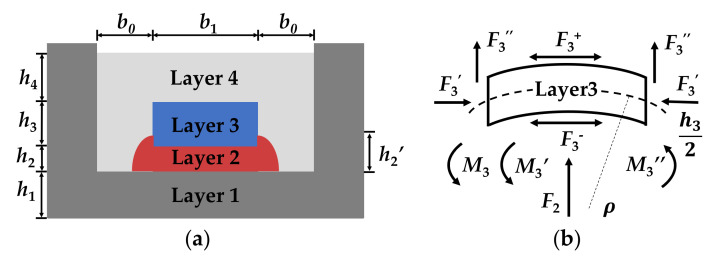
(**a**) Theoretical model of the MEMS pressure sensor; (**b**) mechanical analysis of Layer 3.

Analytic analysis was performed to study output errors of the Wheatstone bridge that are mostly caused by the thermal mismatch between different materials. Figure 4a shows the theoretical model of the MEMS pressure sensor. The structure is simplified into four layers, which respectively correspond to the substrate (Layer 1), bonding adhesive (Layer 2), MEMS chip (Layer 3) and potting adhesive (Layer 4). There are four assumptions in the analytical model: (1) The sensor structure is completely centrosymmetric, to simplify the stress analysis. (2) The material of each layer is homogeneous, isotropic and linearly elastic, to avoid discussing the internal complex stress. (3) The material properties of each layer remain unchanged to simplify the calculation process. Moreover, *h*_i_ is the thickness of each layer, *h*_2__′_ is the thickness of creeping adhesive of Layer 2, *b*_0_ is the width of shield ring and *b*_1_ is the width of center layer. Figure 4b shows the mechanical analysis of Layer 3. There are axial forces caused by horizontal contact layer. *F*_3_^+^ is the force on the upper surface, and *F*_3_^-^ is the force on bottom. *F*_3′_ is the level force caused by shield ring, which prevents the expansion of internal structures. Because the Coefficient of Thermal Expansion (CTE) of potting adhesive is larger than other materials, there are vertical forces *F*_3′_ in the side interface between potting adhesive and MEMS chip. *F*_2_ is the vertical force caused by the expansion of Layer 2. As the layer is bent by multiple stresses, there are multiple torques on the Layer 3. *M*_3_ is the anti-clockwise torque caused by horizontal stresses. *M*_3′_ is the anti-clockwise torque caused by the creeping part of bonding adhesive. *M*_3′_ is the clockwise torque caused by vertical stresses. The curvature radius of Layer 3 is *ρ*. The results show the variation of output error of the Wheatstone bridge at different temperatures, which are compared with experiment and simulation results as shown in Figure 9.

According to the thermal expansion theory of composite structure, the strains of upper and lower surfaces of the connecting layers are equal in values but opposite in directions. It can be described as follows [21]:

(1) $$\varepsilon_{i}^{+}{=\varepsilon }_{i+1}^{-}=\frac{F_{i}{+F}_{i}^{’}}{E_{i}h_{i}b_{1}}{+\alpha }_{i}\Delta t+\frac{h_{i}}{2\rho }=\frac{F_{i+1}{+F}_{i+1}^{’}}{E_{i+1}h_{i+1}b_{1}}{+\alpha }_{i+1}\Delta t-\frac{h_{i+1}}{2\rho }\;(1\;\leq \;i\;\leq \;3)$$

where $\varepsilon_{i}^{+}$ and $\varepsilon_{i+1}^{-}$ are the displacement of upper surface and under surface, respectively; Δ*t* is the difference between loading temperature and room temperature; *E*_i_ is the Young’s modulus; and *α*_i_ is the CTE of each layer. The forces and torques in central structures should be balanced, which leads to the following:

(2) $$\overset{4}{\underset{i=1}{\sum }}F_{i}\left(\overset{i-1}{\underset{j=1}{\sum }}h_{j}+\frac{h_{i}}{2}\right)+\overset{4}{\underset{i=1}{\sum }}M_{i}+\overset{3}{\underset{i=2}{\sum }}(M_{i}^{\prime }-M_{i}^{\prime \prime })=0$$

where $M_{i}^{\prime }$ is the anti-clockwise torque caused by the creeping part of bonding adhesive. $M_{i}^{\prime \prime }$ is the clockwise torque caused by vertical stresses. $M_{i}$, $M_{i}^{\prime }$ and $M_{i}^{\prime \prime }$ can be calculated as follows:

(3) $$M_{i}=\frac{E_{i}h_{i}^{3}}{12\rho }$$

(4) $$M_{2}^{\prime }=\frac{E_{2}E_{4}h_{2}\int_{0}^{h_{2}}\left[\left(2\alpha_{4}-\alpha_{2}\right)(b_{0}-\sqrt{h_{2}^{\prime 2}-h^{2})}+\alpha_{2}\left(b_{0}+b_{1}\right)\right]\left(\frac{h_{2}}{2}-h\right)dh\Delta t}{\left(2E_{2}-E_{4}\right)\int_{0}^{h_{2}}(b_{0}-\sqrt{h_{2}^{\prime 2}-h^{2}})dh+E_{4}(b_{0}+b_{1})h_{2}}$$

(5) $$M_{3}^{\prime }=\frac{2E_{2}E_{3}E_{4}h_{3}\left\{\int_{h_{2}}^{h_{2}^{\prime }}\left[\left(\alpha_{4}-\alpha_{2}\right)\left(b_{0}-\sqrt{h_{2}^{\prime 2}-h^{2}}\right)+\alpha_{2}b_{0}\right]\left(h_{2}+\frac{h_{3}}{2}-h\right)dh+\alpha_{3}h_{3}b_{1}\right\}\Delta t}{2\left(E_{2}E_{3}-E_{3}E_{4}\right)\int_{h_{2}}^{h_{2}^{\prime }}(b_{0}-\sqrt{h_{2}^{\prime 2}-h^{2}})dh+2E_{3}E_{4}b_{0}\left(h_{2}-h_{2}^{\prime }\right)+E_{2}E_{4}h_{3}b_{1}}$$

(6) $$M_{2}^{\prime \prime }=\frac{E_{2}\left(\alpha_{2}-\alpha_{4}\right)\Delta th_{2}b_{1}{}^{2}}{4}$$

(7) $$M_{3}^{\prime \prime }=\frac{E_{3}\left[\left(\alpha_{4}-\alpha_{3}\right),\Delta t\left(h_{2}+h_{3}-h_{2}^{\prime }\right)+\left(\alpha_{2}-\alpha_{3}\right)\Delta t\left(h_{2}^{\prime }-h_{2}\right)\right]b_{1}{}^{2}}{4}$$

where b_1_ is the width of the central structure. Then the stress $F_{3}^{+}\;$ of the upper surface of MEMS can be calculated as follows:

(8) $$F_{3}^{+}=\frac{F_{3}{+F}_{3}^{’}}{h_{3}b_{1}}{+E}_{3}\alpha_{3}\Delta t-\frac{E_{3}h_{3}}{2\rho }$$

## 4. Numerical Simulation

A full-scale geometric model was developed, and finite element simulations were performed, to analyze the effect of the thermal stress of potting adhesive on the output voltage of the MEMS pressure sensor at different thicknesses of potting adhesive (*h*_p_, the distance of the upper surfaces of MEMS chip and potting adhesive).

### 4.1. Geometric Model

**Figure 5:**
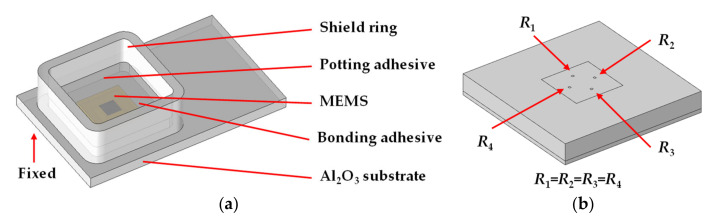
Geometric model of (**a**) MEMS pressure sensor; (**b**) MEMS pressure chip.

The geometric model of the MEMS pressure sensor is shown in Figure 5. It is a full-scale model that contains the key structures of shield ring, potting adhesive, MEMS pressure chip, bonding adhesive and Al_2_O_3_ substrate, as shown in Figure 5a. It has the same geometric data of 18.0 × 11.0 × 4.5 mm with the actual sensor. The MEMS pressure chip is shown in Figure 5b which was simplified by removing the wires and ASIC (Application Specific Integrated Circuit), to reduce the computation cost. Four equivalent piezoresistive resistances are uniformly distributed on the membrane structure with the same orientations. The potting adhesive is designed to cover the MEMS pressure chip, whose *h*_p_ is in the range of 0 to 2.8 mm.

### 4.2. Finite Element Model

**Figure 6:**
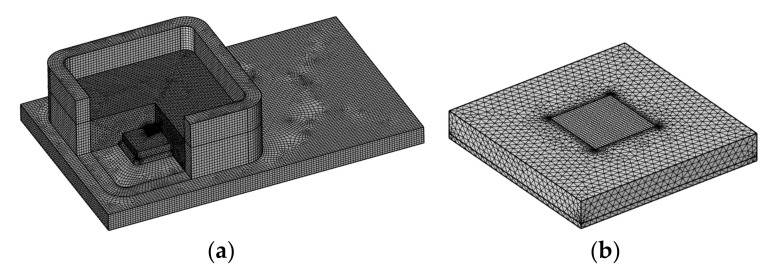
Finite element model of (**a**) MEMS pressure sensor; (**b**) MEMS pressure chip.

Figure 6 shows the finite element model of the MEMS pressure sensor. Figure 6a is the general view of the model, and Figure 6b is a magnification of the refined mesh of the MEMS pressure chip. A multi-scale mesh was used in this model, to reduce the computation cost, which contains 197,074 hexahedra and 835,623 tetrahedra elements. The physical properties of CTE (Coefficient of Thermal Expansion), Young’s modulus, Poisson’s ratio and the density of the MEMS pressure sensor used in the simulation are listed in Table 1. The sensor was fixed at the bottom. The external air pressure applied to the sensor is 0 kPa. The environmental temperature varied from −40 to 150 °C, which is the working temperature range of MEMS pressure sensor. A steady-state solver was used to analyze the stress and strain of MEMS sensor.

### 4.3. Finite Element Simulation

**Figure 7:**
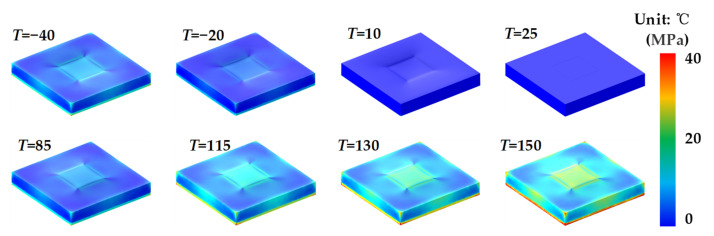
Surface stress of the MEMS pressure chip at different temperatures.

**Figure 8:**
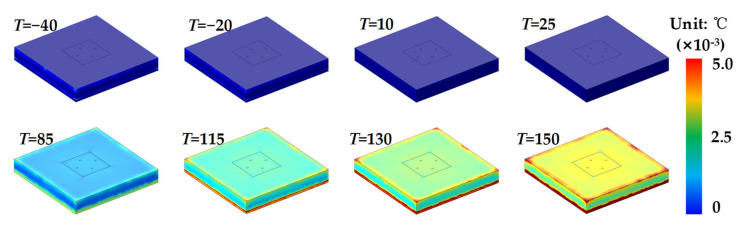
Surface strain of the MEMS pressure chip at different temperatures.

Finite element simulations were carried out by COMSOL 5.5 (COMSOL Inc., Sverige), to study the output error of the MEMS pressure sensor induced by temperature, which varied from −40 to 150 °C. The surface stress displacements of the MEMS pressure chip at different temperatures are shown in Figure 7. It can be found that the thermal stress becomes larger with the temperature increasing from 25 to 150 °C and decreasing from 25 to −40 °C. The stress is mainly concentrated on the areas spliced with the creeping part of bonding adhesive and pressure sensing membrane. The surface strain displacements of the MEMS pressure chip at different temperatures are shown in Figure 8. It can be found that the strain becomes larger with the temperature increasing from 25 to 150 °C, but there is not much strain at low temperatures. The strain is mainly concentrated on the edges of the chip. With the temperature increasing from 25 to 150 °C or decreasing from 25 to −40 °C, the thermal stress on the surface of the MEMS chip becomes larger. That is the reason of interface thermal mismatch. The MEMS chip is made of silicon, and the potting adhesive is made of organic materials. There are big differences of CTE and Young’s modulus between the two materials that lead to thermal stress and strain. The lager the temperature difference, the greater the stress. There are larger stress and strain in the spliced areas with bonding adhesive, which was a result of the larger difference of CTE between bonding adhesive and MEMS chip than potting adhesive and MEMS chip.

**Figure 9:**
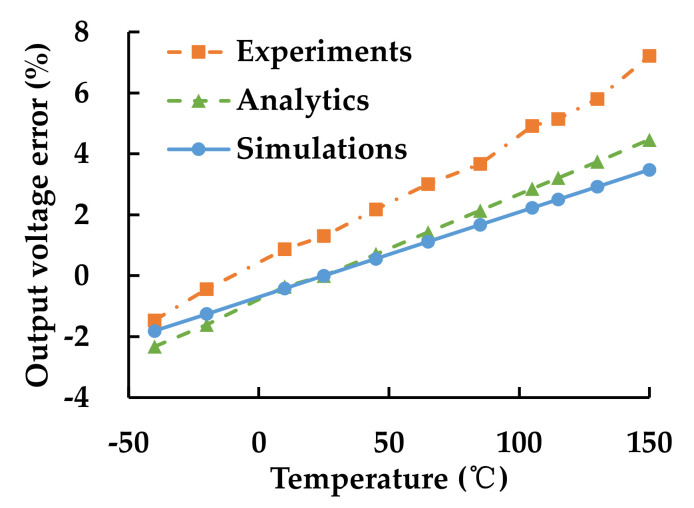
Comparison of the output error of the MEMS pressure sensor, at different temperatures, under 0 kPa pressure.

The strains of the four piezoresistive resistances of the Wheatstone bridge at different temperatures were obtained and calculated to output errors. The comparison of experiment (Figure 3b), analytics (Equations (1)–(8)) and simulation of the output error at different temperatures at 0 kPa pressure is shown in Figure 9. The simulation results agree well with the experiment results. The FE simulation is based on idea model, but there are many uncontrollable factors in actual processes. The process errors caused by asymmetric bonding, crackle on sensing structure or incomplete package were contained in the results of experiment, making the output error of experiments lager than analytics and simulations. The output error of the MEMS pressure sensor was caused by the imbalance of the Wheatstone bridge. The output error is linearly related to temperature and becomes larger when the temperature deviates from the calibrating temperature of 25 °C.

**Figure 10:**
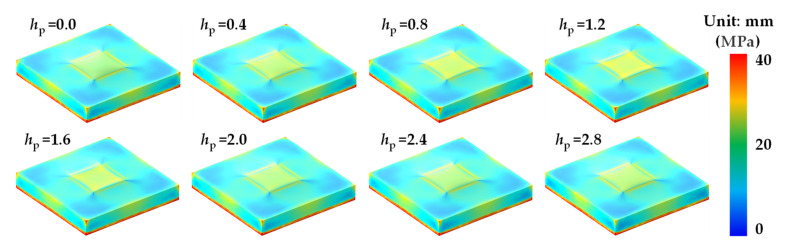
Surface stress of the MEMS pressure chip at different *h*_p_ at 150 °C.

**Figure 11:**
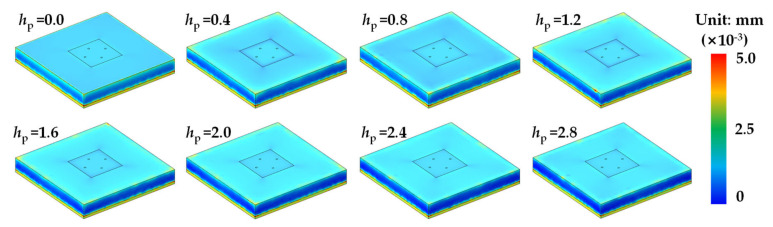
Surface strain of the MEMS pressure chip at different *h*_p_ at 150 °C.

Finite element simulations were carried out, to study the output error of the Wheatstone bridge induced by *h*_p_ which varied from 0 to 2.8 mm. The surface stress and strain displacements of the MEMS chip at different *h*_p_ are shown in Figure 10 and Figure 11, respectively. It can be found that the stresses of sensing structure are larger than adjacent areas. With the increase of *h*_p_, the stresses of sensing structure increase firstly and then decrease. The strains of the upper surface are relatively similar, which are mainly concentrated on the edges and corners.

**Figure 12:**
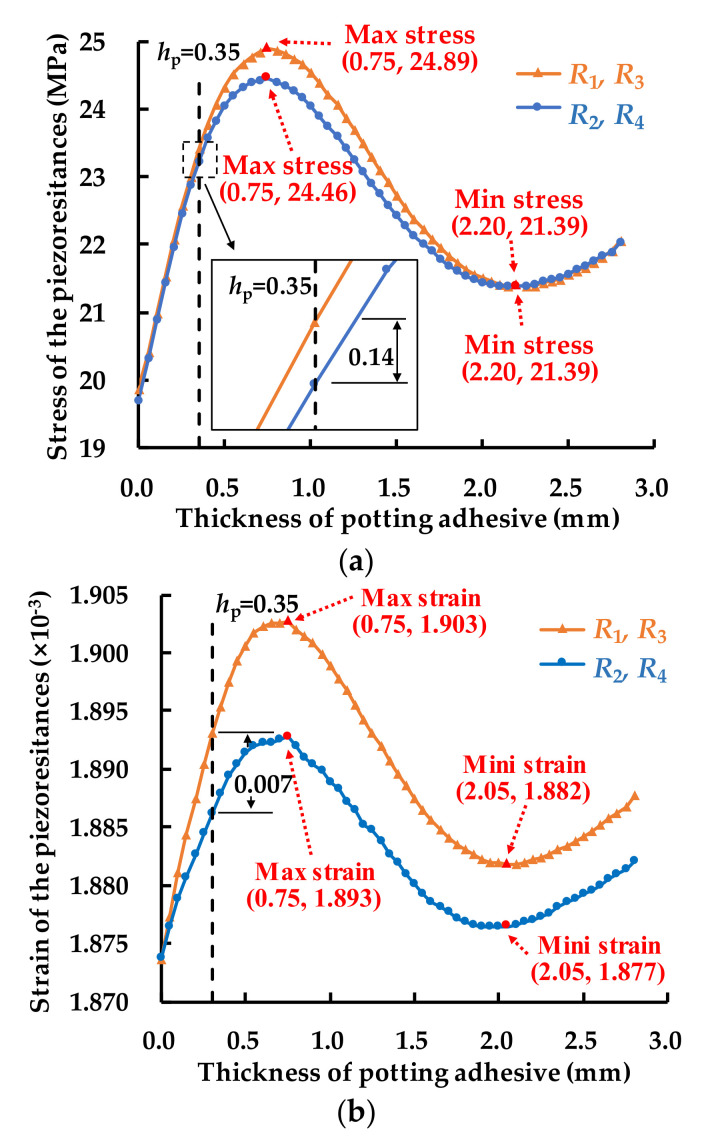

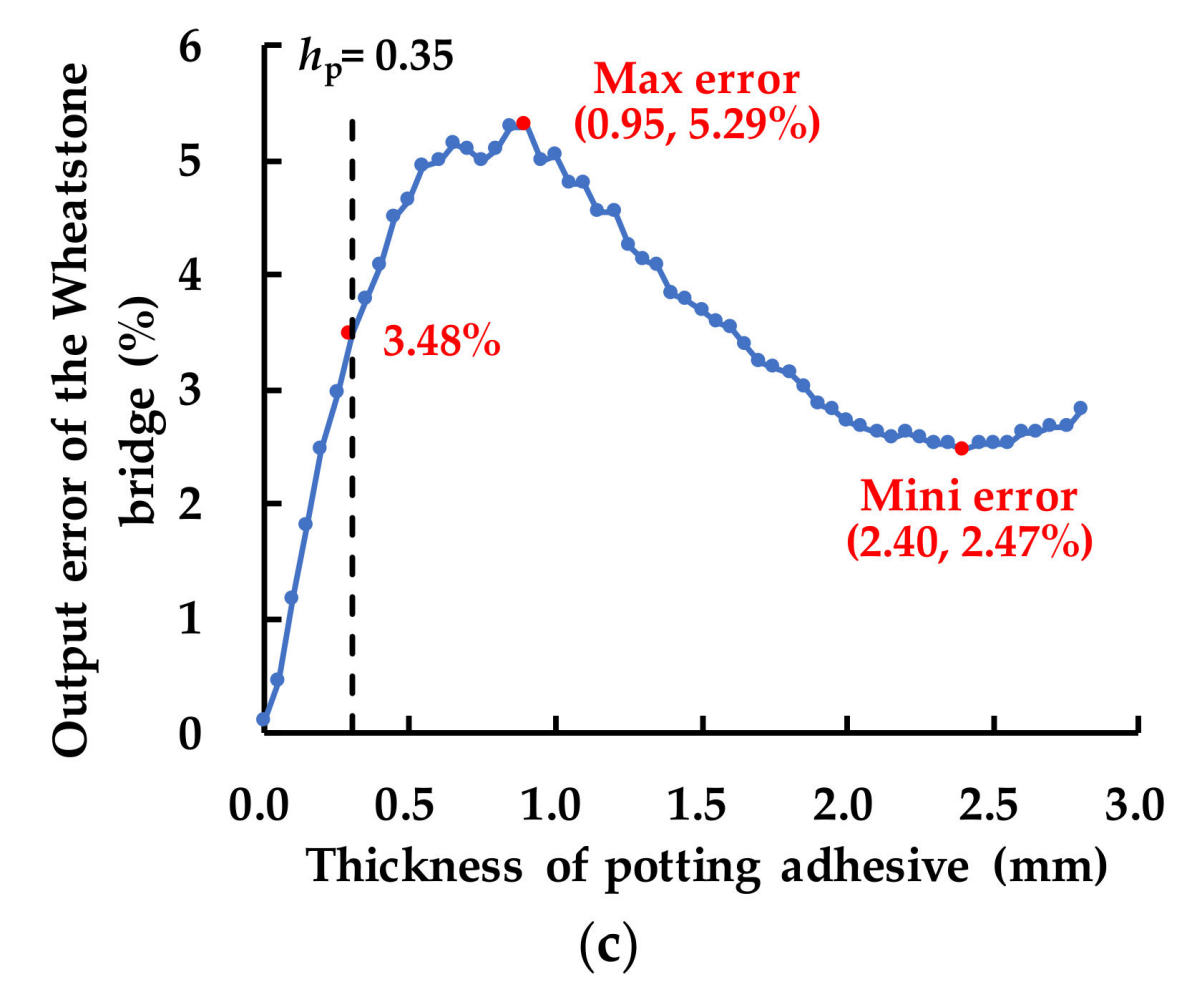
(**a**) Stress and (**b**) strain of the piezoresistances at different *h*_p_ at 150 °C; (**c**) output voltage error of the Wheatstone bridge.

The stress of the piezoresistances at different *h*_p_ are shown in Figure 12a. The changes of *R*1 and *R*3, and *R*2 and *R*4 are separately described in curves which show “N” shape. It can be found that the stresses grow firstly and reach a maximum of 24.89 MPa (*R*_1_, *R*_3_) and 24.46 MPa (*R*_2_, *R*_4_) when *h*_p_ is 0.75 mm. Then the stresses decrease until the *h*_p_ comes to 2.20 mm, where the stresses reach a minimum of 21.39 MPa (*R*_1_, *R*_2_, *R*_3_, *R*_4_). At this *h*_p_, the four piezoresistances in the Wheatstone bridge meet a balance of thermal stress. After that, the stresses grow again with the *h*_p_ increase.

The strain of the piezoresistances at different *h*_p_ is shown in Figure 12b. The changes of *R*1 and *R*3, and *R*2 and *R*4 are separately described in curves which show and N-shape. It can be found that the strains grow firstly and reach a maximum of 1.903 × 10^−3^ (*R*_1_, *R*_3_) and 1.893 × 10^−3^ (*R*_2_, *R*_4_) when *h*_p_ is 0.75 mm. Then the strains decrease until the *h*_p_ comes to 2.05 mm, where the strains reach the minimum of 1.882 × 10^−3^ (*R*_1_, *R*_3_) and 1.877 × 10^−3^ (*R*_2_, *R*_4_). After that, the strains grow again with the *h*_p_ increase.

The output errors of the Wheatstone bridge at different *h*_p_ are shown in Figure 12c. It can be found that the error grows with the *h*_p_ increasing and reaches a maximum of 5.3% when *h*_p_ is 0.9 mm. Then the error decreases until the *h*_p_ comes to 2.40 mm, where the error is 2.47%. After that, the error grows again with the *h*_p_ increase.

It shows a similarity of output error of the Wheatstone bridge in finite element analysis of 3.48% and the thermal stress induced output voltage error in the experiment of 5.90% at the *h*_p_ of 1 mm, at the temperature of 150 °C. The differences between the two results are due to the other process errors in actual MEMS pressure sensors.

## 5. Conclusions

In this study, the potting-adhesive-induced thermal stress in the MEMS pressure sensor was investigated by experiments and finite element analysis. Pressure and temperature experiments were conducted, to test the output voltage variation of the MEMS pressure sensor. Thermal–solid coupling finite element simulations were carried out to study the stress–strain of the MEMS pressure chip, as well as the output error of the Wheatstone bridge. The results between experiments and simulations have a high agreement with each other. It was found that the output error is linearly increasing with the temperature rising or dropping from the calibration temperature. The error varies with the thickness of potting adhesive in an N-shape. The sensor meets the maximum thermal-induced error of 5.29% in the thickness of 0.95 mm and the minimum error of 2.47%, with the thickness increasing to 2.40 mm at 150 °C. This work verifies the feasibility to predict the output error of MEMS pressure sensors by only measuring the thickness of potting adhesive and provides a solution for reducing the output error by strategically adjusting the thickness of the potting adhesive.

## Figures and Tables

**Table 1:** Physical properties of the MEMS pressure sensor.

Structure	Materials	Property (Unit)	Value	Reference
MEMS	Silicon	CTE (1/K)	2.6 × 10^−6^	[22]
		E (GPa)	170	[22]
		μ	0.28	[22]
		ρ (kg/m^3^)	2329	[22]
Potting adhesive	Epoxy resin 1	CTE (1/K)	(14.9~23.0) × 10^−6^	[23]
		E (GPa)	1.30~1.75	[23]
		μ	0.3	[23]
		ρ (kg/m^3^)	2000	[23]
Bonding adhesive	Epoxy resin 2	CTE (1/K)	(26~40) × 10^−6^	[24]
		E (GPa)	2.2~3.5	[24]
		μ	0.3	[24]
		ρ (kg/m^3^)	1700	[24]
Shield ring	Al_2_O_3_	CTE (1/K) E (GPa) μ *ρ* (kg/m^3^)	6.5 × 10^−6^ 400 0.22 3965	[25] [25] [25] [25]
Substrate				
